# Antibacterial properties of cyanoacrylate tissue adhesive: Does the polymerization reaction play a role?

**DOI:** 10.4103/0301-4738.55065

**Published:** 2009

**Authors:** Ivana L Romero, João B N S Malta, Cely B Silva, Lycia M J Mimica, Kaz H Soong, Richard Y Hida

**Affiliations:** 1 Department of Ophthalmology, Santa Casa de São Paulo, São Paulo, Brazil; 2 Department of Microbiology, Santa Casa de São Paulo, São Paulo, Brazil; 3 Department of W.K. Kellogg Eye Center, University of Michigan, Ann Arbor, MI, U.S.A; 4 Department of Ophthalmology, Keio University – School f Medicine, Tokyo, Japan

**Keywords:** Antibacterial, cyanoacrylate, tissue adhesive

## Abstract

**Purpose:**

To ascertain if the polymerization reaction also contributes additionally to the antibacterial effects of two commonly used cyanoacrylate tissue adhesives.

**Materials and Methods:**

Fresh liquid ethyl-cyanoacrylate (EC) and N-butyl-cyanoacrylate (BC) adhesives were applied onto 6-mm sterile filter paper discs. In the first group, the adhesive-soaked discs were immediately placed onto confluent monolayer cultures of bacteria, allowing the polymerization reaction to proceed while in culture. In the second group, the adhesive-soaked disc was allowed to first polymerize prior to being placed onto the bacterial cultures. Four types of bacteria were studied: *Staphylococcus aureus*, *Streptococcus pneumoniae*, *Escherichia coli*, and *Pseudomonas aeruginosa*. Immediately after the discs were applied, the cultures were incubated at 35° C for 24 h. Bacterial inhibitory halos were measured in the cultures at the end of the incubation period.

**Results:**

For EC, exposure of the bacteria to the cyanoacrylate polymerization reaction increased the bacterial inhibitory halos in *Streptococcus pneumonia, Staphylococcus aureus* and *Escherichia coli.* For BC, it increased the bacterial inhibitory halos in *Staphylococcus aureus* and *Streptococcus pneumoniae*. No inhibitory halos were observed in *Pseudomonas aeruginosa.* The bactericidal effect was higher in actively polymerizing EC, compared to previously polymerized EC in *Staphylococcus aureus*, *Streptococcus pneumoniae,* and *Escherichia coli*; however, no such differences were observed for BC.

**Conclusions:**

The polymerization reaction may also be an important factor in the antibacterial properties of EC and BC.

Cyanoacrylate polymers have been used as biological adhesives in the cornea for over 40 years.[1–3] Their monomers are obtained through the condensation of cyanoacetate with formaldehyde in a base-catalyzed reaction.[4] A polymer is formed as a number of monomers join together under the effect of a catalyst, such as water.[5]

The polymerized adhesive promotes wound healing, vascularization, and epithelialization of the injured corneal stroma.[6] It also inhibits corneal melting by directly antagonizing collagenases and by blocking the migration of inflammatory cells, such as polymorphonuclear leukocytes.[4]

The antimicrobial properties of cyanoacrylate tissue adhesives have been reported previously and some authors have even promoted its use in the prophylaxis or treatment of infection in corneal ulcers.[7–9] It has also been postulated that the antimicrobial effects may be derived, at least in part, from the polymerization process itself, although no study has specifically analyzed this assumption.[8]

The aim of the study was to establish the role of the polymerization reaction in conferring additional antibacterial properties to cyanoacrylate tissue adhesives.

## Materials and Methods

Two cyanoacrylate tissue adhesives were studied in the *in vitro* experiments: Ethyl-cyanoacrylate (EC) (Superbonder^®^, Loctite, Brazil) and N-butyl-cyanoacrylate (BC) (Histoacryl^®^, Braun GmbH, Brazil). Six microliters of adhesive were applied onto standard 6-mm sterile filter-paper discs, using sterile micropipettes (Eppendorf^®^, Hamburg, Germany) under sterile conditions.

The following bacterial strains from the American type culture collection (ATCC) were analyzed: *Staphylococcus aureus* (ATCC25924), *Streptococcus pneumoniae* (ATCC49619), *Escherichia coli* (ATCC25922), and *Pseudomonas aeruginosa* (ATCC27853). They were primarily incubated in a nutrient broth at a temperature of 35°C until reaching 0.5 on the McFarland scale (turbidity of bacterial suspension at a population of approximately 1.5 × 10^8^ organisms). The bacteria were then transferred as confluent monolayer cultures to Müller-Hinton media following the Kirby-Bauer modified technique, with the exception of *Streptococcus pneumoniae*, which was transferred to blood agar media.

Ten Müller-Hinton agar plates (150-mm diameter) were used for each of the following bacteria: *Staphylococcus aureus*, *Escherichia coli*, and *Pseudomonas aeruginosa*. Twenty blood agar plates (90-mm diameter) were used for *Streptococcus pneumoniae*. A blank disc without adhesive was placed in the center of each bacterial plate as a control.

**Figure 1:**
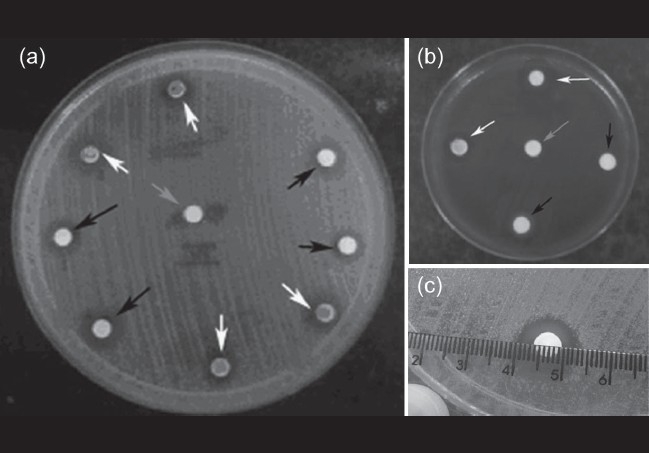
(a) Müller-Hinton media plate cultured with *Staphylococcus aureus*. (b) Blood agar plate cultured with *Streptococcus pneumoniae*. Inhibition halos seen around the discs of ethyl-cyanoacrylate (EC) (black arrows) and N-butyl-cyanocrylate (BC) (white arrows). Central disc is control (gray arrow). (c) Measurement of inhibition halo with ruler

In each Müller-Hinton plate, eight additional discs were placed onto the cultures (in addition to the control disc): two with actively polymerizing (liquid) EC (EC-liq); two with previously polymerized (solidified) EC (EC-poly); two with actively polymerizing (liquid) BC (BC-liq); and two with previously polymerized (solidified) BC (BC-poly). For the blood agar plates, four discs were placed onto the cultures (in addition to the control disc): one with actively polymerizing (liquid) EC (EC-liq); one with previously polymerized (solidified) EC (EC-poly); one with actively polymerizing (liquid) BC (BC-liq); and one with previously polymerized (solidified) BC (BC-poly). To achieve prior polymerization of the cyanoacrylate in EC-poly and BC-poly, the adhesive-soaked discs were exposed to air under a sterile hood for 30 min before being placed on the cultures. All actively polymerized samples were applied directly into the disc placed in the plate. All plates were then incubated at 35°C for 24 h, after which the bacterial inhibitory halos, if present, were measured in millimeters [Fig. 1].

In order to evaluate if the bacterial inhibitory halos were the result of mere bacteriostasis or actual bactericidal effects, two samples per halo were collected from the clear agar within the inhibitory halos and re-cultured on new bacterial culture plates. The new plates were re-incubated at 35°C and analyzed after 48 h. Bactericidal activity was measured by calculating the percentage of plates with no bacterial growth. All the microbiological procedures and readings were performed by one author (CBS). The results were statistically analyzed with the Student T-test and significance was defined as *P* < 0.05.

## Results

Table 1 shows the mean and standard deviation (SD) of the inhibitory halo (mm) for EC-liq and EC-poly for each microorganism studied. For EC, the polymerization reaction appeared to enhance the antibacterial effect in *Streptococcus pneumonia* (*P*=0.0014), *Escherichia coli* (*P*<0.0001) and *Staphylococcus aureus* (*P*=0.019), but not in *Pseudomonas aeruginosa*.

**Table 1:** Mean and standard deviation (SD) of inhibitory halos (mm), comparing actively polymerizing liquid ethyl-cyanoacrylate discs (EC-liq) with previously polymerized ethyl-cyanoacrylate discs (EC-poly) for *Staphylococcus aureus, Streptococcus pneumoniae, Escherichia coli*, and *Pseudomonas aeruginosa*. Statistical significance of difference between EC-liq and EC-poly is given as P-values

	*Staphylococcus aureus*		*Streptococcus pneumoniae*		*Escherichia coli*		*Pseudomonas aeruginosa*	
				
	EC-liq	EC-poly	EC-liq	EC-poly	EC-liq	EC-poly	EC-liq	EC-poly
Mean	14.05	13.45	14.25	12.70	11.2	0	0	0
SD	1.00	1.00	1.33	1.38	2.67	0	0	0
*P*		0.019		0.0014		<0.0001		N/A

Table 2 shows the mean and SD of the inhibitory halo (mm) for BC-liq and BC-poly for each microorganism studied. For BC, the polymerization reaction appeared to enhance the antibacterial effect in *Staphylococcus aureus* (*P*<0.0001) and *Streptococcus pneumoniae* (*P*=0.0010), but not in *Escherichia coli* and *Pseudomonas aeruginosa*.

**Table 2:** Mean and standard deviation (SD) of inhibitory halos (mm), comparing actively polymerizing liquid N-butylcyanoacrylate discs (BC-liq) with previously polymerized N-butyl-cyanoacrylate discs (BC-poly) for *Staphylococcus aureus, Streptococcus pneumoniae, Escherichia coli*, and *Pseudomonas aeruginosa*

	*Staphylococcus aureus*		*Streptococcus pneumoniae*		*Escherichia coli*		*Pseudomonas aeruginosa*	
	
	BC-liq	BC-poly	BC-liq	BC-poly	BC-liq	BC-poly	BC-liq	BC-poly
			
Mean	13.40	11.70	14.70	12.75	0	0	0	0
SD	1.05	0.86	2.13	1.25	0	0	0	0
*P*		<0.0001		0.0010		N/A		N/A

Statistical significance of difference between BC-liq and BC-poly is given as *P*-values.

The results of the bactericidal analyses of EC and BC are summarized in Table 3. The bactericidal effect was higher in EC-liq when compared to EC-poly in *Staphylococcus aureus*, *Streptococcus pneumoniae*, and *Escherichia coli*, but this bactericidal enhancement by the polymerization reaction was not observed in BC. The bactericidal effect was not analyzed for *Pseudomonas aeruginosa*, since there was no inhibitory halo.

**Table 3:** Bactericidal effect of actively polymerizing liquid ethyl-cyanoacrylate (EC) and N-buthyl-cyanoacrylate (BC) (EC-liq and BC-liq) and previously polymerized (EC-poly and BC-poly) against the microorganisms in question

	EC-liq	EC-poly	BC-liq	BC-poly
*Staphylococcus aureus*	20%	10%	0%	10%
*Streptococcus pneumoniae*	70%	40%	70%	70%
*Escherichia coli*	30%	0%	0%	0%
*Pseudomonas aeruginosa*	0%	0%	0%	0%

## Discussion

Antibacterial effects were enhanced by the polymerization reaction in EC for *Streptococcus pneumonia*, *Staphylococus aureus* and *Escherichia coli*. In *Escherichia coli*, however, inhibitory halos were totally absent without exposure to the active polymerization reaction. For BC, inhibitory halos were observed only for gram-positive bacteria. No inhibitory halo was observed for *Pseudomonas aeruginosa* for either EC or BC, with or without exposure to the active polymerization reaction.

The antibacterial effects of cyanoacrylate are greater in gram-positive bacteria than in gram-negative, possibly because the latter are protected by an outer carbohydrate capsule.[10] Our results did indeed show that the susceptibility to cyanoacrylate of the two gram-positive organisms tested, *Staphylococcus aureus* and *Streptococcus pneumoniae*, to be much greater than of the two gram-negative organisms tested, *Escherichia coli* and *Pseudomonas aeruginosa*. Eiferman *et al*. reported the absence of inhibitory halos in *Klebsiella pneumoniae*[7] a gram-negative enterobacterium similar to *Escherichia coli*.

EC appeared to have a greater antibacterial effect than BC for *Staphylococcus aureus* and *Escherichia coli*. This difference may be due to the greater tissue toxicity and antimicrobial effects associated with shorter-alkyl chain cyanoacrylates, such as EC, over the longer-alkyl chain cyanoacrylates, such as BC. As a rule, shorter alkyl-chain cyanoacrylates are characterized by greater instability and faster chemical degradation than the longer alkyl-chain counterparts, resulting in higher levels of ambient degradation, products such as cyanoacetate and formaldehyde.[11]

The bactericidal effect of the adhesives was lower than in previous studies.[7–9,12] This discrepancy may be due in part to differences in volumes of cyanoacrylate studied, as well as the unstandardized volumes utilized in previous studies. Our use of standard diameter filter-paper disc carriers instead of merely dropping the cyanoacrylate directly onto the cultures could contribute to this discrepancy from previous studies. The diameter of the cyanoacrylate in the free-drop method in previous studies may be much more variable than that of our standardized disc method. Although Eiferman *et al*. established a standard volume of adhesive of 100 μl, no bactericidal studies were performed.[7]

A polymer of cyanoacrylate is formed as a number of monomers join together under the effect of any catalyst (water). The polymer decomposes to produce cyanoacetate and formaldehyde. Such degradation products can diffuse out. Therefore, there is an inhibition halo even in a polymerized state.[4,5]

Although the effect of the polymerization reaction was relatively small for gram-positive micro organisms, the values were statistically significant. Nevertheless, as per gram-negative micro organism like *Escherichia coli*, the effect of the polymerization reaction in EC was very important.

Regarding restrictions of this study, “*in vivo*” reproduction is not possible because the material loses its adhesive capacity to mold onto irregular surfaces after polymerization reaction. On top of that, when cyanoacrylate-based tissue adhesive gets into contact with water, it solidifies. Therefore, it is not possible to use different concentrations of the adhesive and to obtain the MIC 90, which is used to evaluate antimicrobial properties in antibiotics and is defined as the antimicrobial concentration that inhibits growth of 90% of the microorganisms.

In conclusion, the polymerization reaction appears to have an important contributory role in the antibacterial activity of cyanoacrylate tissue adhesives and may be exploited in the treatment of corneal ulcers. Therefore, in severe or recalcitrant gram-positive bacterial corneal ulcers, for instance, one may consider the use of shorter alkyl-chain adhesives with their strong antibacterial properties, as a supplement to topical antibiotics. This antibacterial effect may be an advantage when glue is applied to the cornea in patients with melting or perforation.
